# Dissociating the time courses of the cross-modal semantic priming effects elicited by naturalistic sounds and spoken words

**DOI:** 10.3758/s13423-017-1324-6

**Published:** 2017-06-09

**Authors:** Yi-Chuan Chen, Charles Spence

**Affiliations:** Crossmodal Research Laboratory, Department of Experimental Psychology, University of Oxford, 9 South Parks Road, Oxford, OX1 3UD UK

**Keywords:** Semantic, Multisensory, Audiovisual, Sensitivity, Priming

## Abstract

The present study compared the time courses of the cross-modal semantic priming effects elicited by naturalistic sounds and spoken words on visual picture processing. Following an auditory prime, a picture (or blank frame) was briefly presented and then immediately masked. The participants had to judge whether or not a picture had been presented. Naturalistic sounds consistently elicited a cross-modal semantic priming effect on visual sensitivity (*d'*) for pictures (higher *d'* in the congruent than in the incongruent condition) at the 350-ms rather than at the 1,000-ms stimulus onset asynchrony (SOA). Spoken words mainly elicited a cross-modal semantic priming effect at the 1,000-ms rather than at the 350-ms SOA, but this effect was modulated by the order of testing these two SOAs. It would therefore appear that visual picture processing can be rapidly primed by naturalistic sounds via cross-modal associations, and this effect is short lived. In contrast, spoken words prime visual picture processing over a wider range of prime-target intervals, though this effect was conditioned by the prior context.

In daily life, hearing the sound of a dog barking is likely informative with regard to the identity of a creature that is glimpsed, albeit briefly (Chen & Spence, 2010). Indeed, the presentation of either a naturalistic sound or spoken word enhances the sensitivity (*d'*) of visual object detection (Chen & Spence, 2011; Lupyan & Ward, 2013). Such results suggest that the meaning of the auditory cue facilitates visual processing and boosts the breakthrough of the visual stimulus into awareness cross-modally rather than simply giving rise to some sort of criterion change (note that the dog barking certainly induces a likely guess that the creature might be a dog as well).

The time courses of cross-modal semantic priming effects, however, appear to be different for naturalistic sounds and spoken words. Chen and Spence (2011) demonstrated that when leading the target picture by 346 ms, only naturalistic sounds (rather than spoken words) elicited a semantic priming effect on visual picture sensitivity in a simple detection task (when judging whether a picture was present or not). These results were explained based on evidence suggesting that naturalistic sounds access their associated meaning faster than spoken words do (Chen & Spence, 2013; Cummings et al., 2006; Saygin, Dick, & Bates, 2005). The different processing times plausibly stem from the differing routes of semantic access for each type of auditory stimulus: Naturalistic sounds access semantic information directly, whereas spoken words have to access their meanings via lexical representations (Barsalou, Santos, Simmons, & Wilson, 2008; Chen & Spence, 2011; Glaser & Glaser, 1989).

Lupyan and colleagues, on the other hand, demonstrated an advantage for spoken words over naturalistic sounds at longer SOAs (around 1,000 ms or more; Edmiston & Lupyan, 2015; Lupyan & Thompson-Schill, 2012 1). The participants in their studies had to verify whether the auditory cue (either a naturalistic sound or a spoken word) and the subsequently presented picture matched or not. The results demonstrate that the participants’ reaction times (RTs) were shorter for spoken words than for naturalistic sounds. Further evidence comes from an event-related potential (ERP) study: When a spoken word led a target picture by around 1,670 ms, the P1 component associated with the picture (at 70–125 ms after onset) occurred earlier in the congruent than in the incongruent condition, but no such congruency effect was induced by naturalistic sounds (Boutonnet & Lupyan, 2015). These results were explained in terms of spoken words being associated with semantic representations that are more abstract and categorical, thus providing a conceptual cue regarding a given object that is general rather than specific to a particular exemplar, as compared to naturalistic sounds (Edmiston & Lupyan, 2015; Lupyan & Thompson-Schill, 2012).

Given the different SOAs and methods, and given the different mechanisms proposed by previous research (Chen & Spence, 2011; Edmiston & Lupyan, 2015; Lupyan & Thompson-Schill, 2012), we wanted to carefully examine the time courses of cross-modal semantic priming effects elicited by naturalistic sounds and spoken words. Two critical SOAs were chosen: The 350- ms SOA is close to the interval at which Chen and Spence (2011) demonstrated cross-modal semantic priming by naturalistic sounds (but not by spoken words) in a picture detection task. The 1,000-ms SOA (the interstimulus interval, ISI, was 500–650 ms) corresponds to the ISI somewhere between 400 and 1,000 ms used by Lupyan and Thompson-Schill (2012), the conditions demonstrated a cross-modal semantic advantage for spoken words over naturalistic sounds. In Experiment 1, each participant was tested with only one of the SOAs, following the designs of Chen and Spence (2011) and Lupyan and Thompson-Schill (2012). In Experiment 2, participants were tested with both SOAs in a counterbalanced order. In this case, we further examined whether the time courses of cross-modal semantic priming effects are stable or modulated by prior context.

## Experiment 1

### Method

#### Participants

Forty volunteers (10 males, mean age 22.2 years) took part in this experiment in exchange for course credit or five pounds (UK sterling). The participants were native English speakers or bilinguals who had started to learn English by 5 years of age. All participants had normal or corrected-to-normal vision and normal hearing by self-report, and all were naïve as to the purpose of the study. Written informed consent was obtained prior to the start of the study. The study was approved by the Medical Sciences Inter Divisional Research Ethics Committee, University of Oxford (MSD-IDREC-C1-2014-143).

#### Apparatus and stimuli

The visual stimuli were presented on a 23-inch LED monitor controlled by a personal computer. The participants sat at a viewing distance of 58 cm from the monitor in a dimly lit chamber. Twenty-four outline-drawings (12 living and 12 nonliving things) taken from Snodgrass and Vanderwart (1980) and Bates et al. (2003), as well as their mirror images, were used as visual targets (see Appendix). Five pattern masks were created by overlapping 20 nonobject figures randomly selected from Magnié, Besson, Poncet, and Dolisi (2003). Each pattern covered an area of 5.9° × 5.9°, sufficient to completely occlude all of the target pictures.

The auditory stimuli (8 bit mono; 22500 Hz digitization) were presented over closed-ear headphones and ranged in loudness from 31 to 51 dB sound pressure level (SPL). The naturalistic sounds were those produced by each of the objects. The spoken words consisted of the most commonly agreed-upon name used to refer each picture (Bates et al. 2003; Snodgrass & Vanderwart, 1980) and were produced by a female native English speaker. The naturalistic sound and the spoken word associated with the same picture were edited to have the same duration. The root mean square values of all of the auditory stimuli were equalized.

#### Design

Two within-participants factors, prime type (naturalistic sound or spoken word) and congruency (congruent or incongruent), and one between-participants factor, SOA (350 or 1,000 ms), were manipulated. Naturalistic sounds and spoken words were presented in separate blocks of trials. Congruent and incongruent trials were mixed within blocks: The auditory cue matched the picture in the congruent trials, but they belonged to different categories based on the fundamental living thing versus nonliving thing separation in the incongruent trials. Each SOA was tested with 20 participants.

All 24 pictures and their mirror images were presented once in each block—either one was presented in the congruent trial and the other in the incongruent trial (and they were swapped in another block). These trials were used to estimate the participant’s hit rate in the congruent and incongruent conditions, respectively. An additional 48 picture-absent trials, consisting of an auditory cue and a blank frame, were presented to estimate the participant’s false alarm (FA) rate. These 96 trials were presented in a completely randomized order. There were two blocks for both naturalistic sounds and spoken words, and the order of these two types of auditory stimuli was counterbalanced across participants. The participants were not given any information concerning the possible semantic congruency between the auditory cue and picture prior to taking part in the study.

#### Procedure

**Fig. 1:**
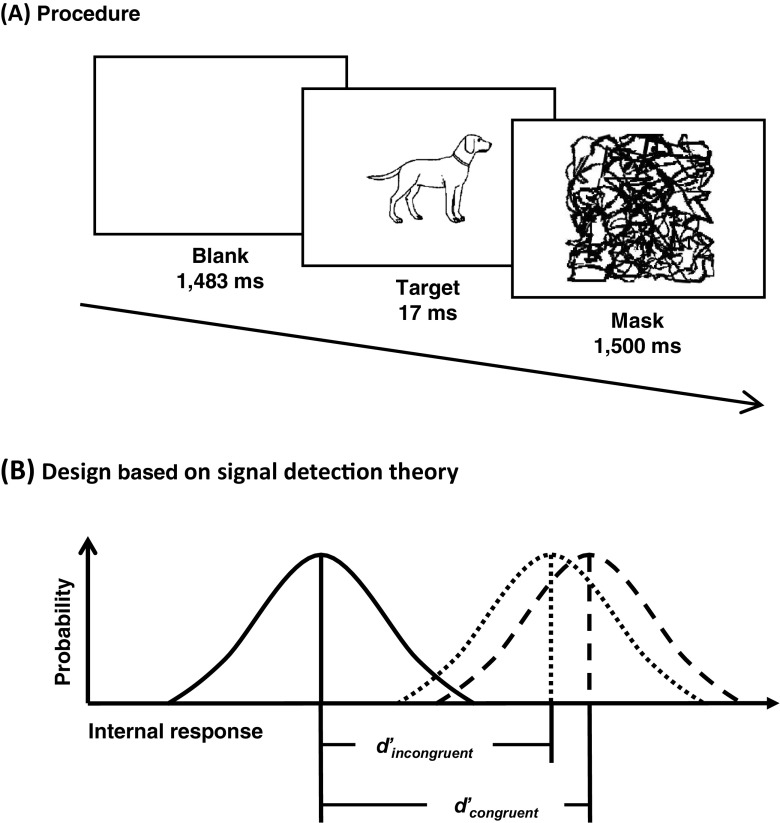
**a** Sequence of three frames presented in each trial: A blank, a target picture (e.g., a dog), and a pattern mask. The target picture and pattern mask were presented in black in the center of a white background. **b** Represents the current experimental design in terms of signal detection theory. The distributions of *dashed*, *dotted*, and *solid lines* represent target present/congruent, target present/incongruent, and target absent conditions, respectively. In this design, the congruent and incongruent conditions share the same FA rate. The sensitivity (*d'*) was calculated using the equations: *d'* = *z*(hit rate) – *z*(FA rate) in the congruent and incongruent conditions, separately (Green & Swets, 1966; Macmillan & Creelman, 2005)

The participants initiated a block of trials by pressing the enter key on the keyboard in front of them. In each trial (see Fig. 1a), a blank frame was followed by either a frame with a picture or another blank for 17 ms (one frame at the screen refresh rate of 60 Hz). The pattern mask was presented immediately thereafter; meanwhile, the participants had to decide whether they had seen a picture (irrespective of its identity) presented before the mask by pressing the space bar. The participants were informed that the task was not speeded, and they should only respond if they were sure that they had seen a picture (i.e., they should maintain a strict response criterion).

Prior to the start of the main experiment, all of the pictures and their matched names were presented on the monitor in a completely randomized order across participants. Each picture-name pair was presented for 1,500 ms and interleaved by a blank frame for 500 ms. An easy practice session (eight trials with a picture duration of 33 ms) and a harder practice session (16 trials with a picture duration of 17 ms) were conducted prior to the main experiment. In the easy practice session, the accuracy had to reach 85%, or it was repeated up to three times. The stimuli in the practice session were not used in the main experiment. The experiment lasted for approximately 30 minutes.

#### Results

**Fig. 2:**
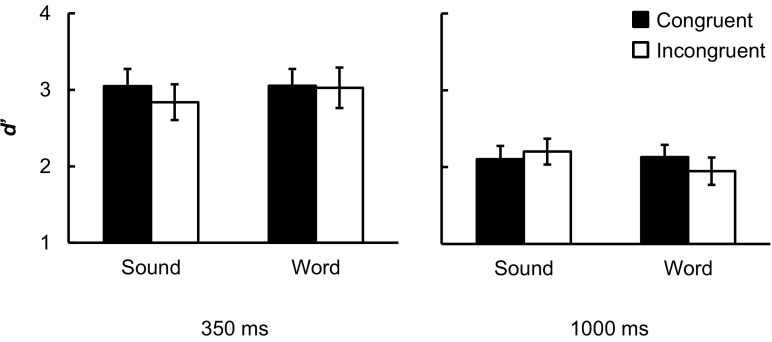
Mean sensitivity (*d'*) at the 350- and 1,000-ms SOAs in Experiment 1. *Error bars* indicate ±1 *SEM*. Sound = naturalistic sounds; Word = spoken words

For both naturalistic sounds and spoken words, the hit rate in the congruent and incongruent conditions was estimated on the basis of 48 trials (24 pictures × 2 blocks), while the FA rate was estimated on the basis of 96 trials (48 picture-absent trials × 2 blocks; see Table 1); *d'* values were calculated based on the hit and FA rate (see Figs. 1b–2), and then submitted to a three-way analysis of variance (ANOVA) with the factors of congruency, prime type, and SOA (see Table 2 for results). Critically, there was a significant three-way interaction. Paired *t* tests (Holm-Bonferroni correction, one-tailed were used because higher *d'* in the congruent than the incongruent condition was expected) demonstrated the congruency effect by naturalistic sounds at the 350-ms SOA, *t*(19) = 2.81, *p* < .05, but not at the 1,000-ms SOA, *t*(19) = -1.63, *p* = .12; in contrast, the congruency effect by spoken words occurred at the 1,000-ms SOA, *t*(19) = 2.87, *p* < .05, but not at the 350-ms SOA, *t*(19) = 0.32, *p* = .75. We therefore replicated the results at the 350-ms SOA reported in Chen and Spence (2011).

**Table 1:** Percentage of hit and false alarm (FA) rates (*SE* in parentheses) in each of the conditions in Experiment 1

SOA (ms)	Sound type	Hit rate		FA rate
		Congruent	Incongruent	
350	Naturalistic sound	83.4 (3.8)	79.1 (4.0)	7.1 (2.6)
	Spoken word	84.0 (3.6)	81.9 (4.6)	6.7 (2.1)
1,000	Naturalistic sound	65.3 (3.7)	68.3 (4.2)	10.3 (4.0)
	Spoken word	73.8 (3.5)	67.3 (3.9)	12.4 (3.6)

**Table 2:** Results of analysis of sensitivity (*d'*) in Experiment 1 (three-way ANOVA: Congruency × Prime Type × SOA)

Effect	*F*(1, 38)	*p*	η_p_^2^	Note
Congruency	5.92	<.05	0.14	Congruent (2.58) > Incongruent (2.50)
SOA	11.22	<.005	0.23	350 ms (2.99) > 1,000 ms (2.10)
Congruency × Prime Type × SOA	9.99	<.005	0.21	

## Experiment 2

### Method

Thirty-six volunteers (seven males, mean age 19.7 years) took part in this experiment. Three factors were tested with all participants: prime type (naturalistic sound or spoken word), congruency (congruent or incongruent), and SOA (350 or 1,000 ms). The fourth factor, the order in which the SOAs were tested, was manipulated between participants: Half of the participants were tested with the 350-ms SOA in the first session and the 1,000-ms SOA in the second session (Group 1: 350–1,000 ms); the order was reversed for the remainder of the participants (Group 2: 1,000–350 ms). The stimuli and task were the same as in Experiment 1. The experiment took an hour to complete.

### Results

**Fig. 3:**
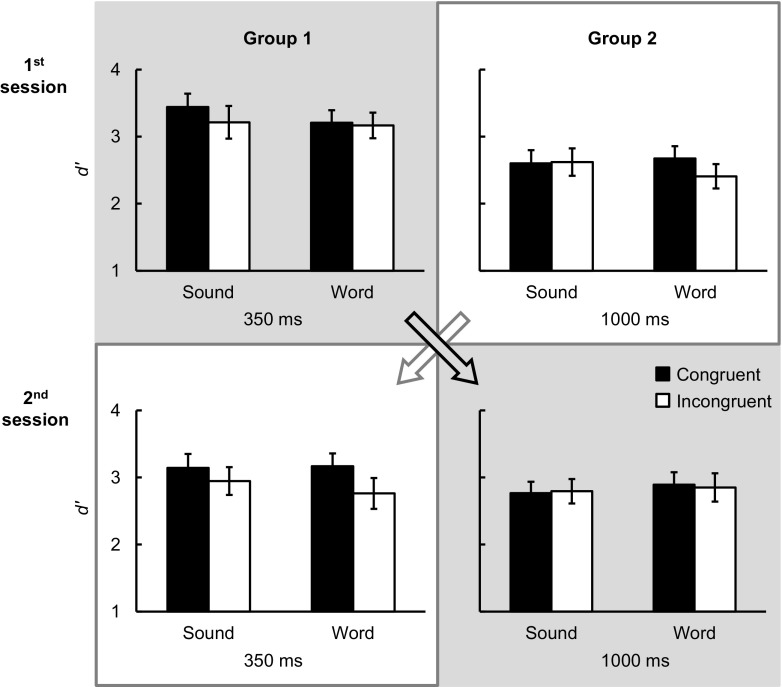
Mean sensitivity (*d'*) at the 350- and 1,000-ms SOAs for Group 1 (tested in the order 350- and then 1,000-ms SOA) and Group 2 (with the order reversed) in Experiment 2. *Error bars* indicate ±1 *SEM*. Sound = naturalistic sounds; Word = spoken words

The participant’s *d'* (see Fig. 3) was calculated based on the hit and FA rates in each condition (see Table 3), and then submitted to a four-way ANOVA (see Table 4a for the results). There was a significant three-way interaction between congruency, prime type, and order. Two separate two-way ANOVAs for each prime type with the factors of congruency and order demonstrated that the congruency effect was significant for naturalistic sounds, *F*(1, 34) = 7.50, *p* < .05, η_p_^2^ = 0.18, without being modulated by order (Congruency × Order: *F* < 1, *p* = .87, η_p_^2^ = 0.001). However, for spoken words, the congruency effect was modulated by order (Congruency × Order): *F*(1, 34) = 12.41, *p* < .005, η_p_^2^ = 0.27. Post hoc tests demonstrated that the congruency effect by spoken words was significant in Group 2 (1,000–350 ms), *t*(17) = 6.18, *p* < .001, but not in Group 1 (350–1,000 ms), *t*(17) = 0.65, *p* = .53. These results therefore suggest that the SOA order influenced the cross-modal semantic congruency effect elicited by spoken words but not by naturalistic sounds. Such a carryover effect of the SOA from one session to the next may mask the modulation of SOA on the congruency effect from auditory cues. The data from the two sessions were therefore analyzed separately.

**Table 3:** Percentage of hit and false alarm (FA) rates (*SE* in parentheses) in each of the conditions in Experiment 2

	SOA (ms)	Sound type	Hit rate		FA rate
			Congruent	Incongruent	
Group 1	350 (first session)	Naturalistic sound	88.0 (2.3)	81.0 (4.3)	3.2 (1.4)
		Spoken word	82.6 (3.8)	81.4 (4.0)	2.2 (0.6)
	1,000 (second session)	Naturalistic sound	71.9 (3.7)	72.3 (4.1)	2.5 (0.9)
		Spoken word	71.4 (4.4)	69.0 (5.2)	1.4 (0.4)
Group 2	350 (second session)	Naturalistic sound	80.7 (4.0)	75.8 (5.1)	5.6 (3.3)
		Spoken word	79.2 (3.8)	67.4 (5.4)	3.5 (2.2)
	1,000 (first session)	Naturalistic sound	69.4 (4.0)	69.7 (4.5)	4.7 (2.2)
		Spoken word	71.1 (4.9)	64.0 (5.9)	5.5 (2.3)

**Table 4:** Results of analysis of sensitivity (*d'*) in Experiment 2

Effect	*F*(1, 34)	*p*	η_p_^2^	Note
(A) Four-way ANOVA (Congruency × Prime Type × SOA × Order)				
Congruency	17.85	<.001	0.34	Congruent (2.99) > Incongruent (2.85)
SOA	45.32	<.001	0.57	350 ms (3.13) > 1,000 ms (2.70)
Congruency × SOA	10.15	<.005	0.23	
Congruency × Prime Type	6.46	<.05	0.16	
Congruency × Order	4.49	<.05	0.12	
Congruency × Prime Type × Order	16.95	<.001	0.33	
(B) First session: three-way ANOVA (Congruency × Prime Type × SOA)				
Congruency	8.85	<.01	0.21	Congruent (2.98) > Incongruent (2.85)
SOA	6.65	<.05	0.16	350 ms (3.26) > 1,000 ms (2.58)
Congruency × Prime Type × SOA	12.24	<.005	0.27	
(C) Second session: three-way ANOVA (Congruency × Prime Type × SOA)				
Congruency	15.83	<.001	0.32	Congruent (2.99) > Incongruent (2.84)
Congruency × SOA	14.41	<.005	0.30	
Congruency × Prime Type	4.96	<.05	0.13	

When only including the data from the first session (top row in Fig. 3), a three-way ANOVA with the factors of congruency, prime type, and SOA (with SOA as a between-participant factor) was conducted (see Table 4b). This is the same design as in Experiment 1, and the results were replicated: The three-way interaction was significant. Paired t tests demonstrated that the congruency effect by naturalistic sounds was only observed at the 350-ms SOA, *t*(17) = 2.50, *p* < .05, but not at the 1,000-ms SOA, *t*(17) = -0.27, *p* = .79. In contrast, the congruency effect by spoken words was only statistically significant at the 1,000-ms SOA, *t*(17) = 3.94, *p* < .005, but not at the 350-ms SOA, *t*(17) = 0.50, *p* = .62.

The results of the second session (bottom row in Fig. 3) were different from the first session (see Table 4c). The significant interaction between congruency and SOA was attributed to the fact that the congruency effect was only significant at the 350-ms SOA, *t*(17) = 4.69, *p* < .001, but not at the 1,000-ms SOA, *t*(17) = 0.16, *p* = .87. Planned comparisons demonstrated that the congruency effect was significant at the 350-ms SOA for both naturalistic sounds, *t*(17) = 2.98, *p* < .05, and spoken words, *t*(17) = 4.30, *p* < .001, but for neither at the 1,000-ms SOA, *t*(17) = -0.57, *p* = .58, and *t*(17) = 0.65, *p* = .53, respectively. The significant interaction between congruency and prime type reflected the congruency effect being significant for spoken words, *t*(35) = 3.48, *p* < .005, but only marginally significant for naturalistic sounds, *t*(35) = 1.88, *p* = .07. The latter perhaps results from the slightly higher *d'* in the incongruent than in the congruent condition at the 1,000-ms SOA.

## General discussion

The results of the present study demonstrate that the presentation of naturalistic sounds enhanced the visual sensitivity of semantically congruent pictures at the shorter SOA (350 ms) than spoken words did (1,000 ms) when each participant just encountered either one of the SOAs. The cross-modal semantic priming effects elicited by the presentation of naturalistic sounds versus spoken words can therefore be dissociated in terms of their differing time courses. Furthermore, naturalistic sounds consistently primed the visual pictures at the short SOA; in contrast, the priming effect elicited by spoken words was significantly modulated by the SOA tested beforehand. Specifically, when the 1,000-ms SOA (demonstrating a significant priming effect) was tested first, the priming effect carried over to the 350-ms SOA (this was not observed in Experiment 1). However, when the 350-ms SOA (where no priming effect was observed) was tested first, the priming effect at the 1,000-ms SOA was eliminated as well. Finally, higher sensitivity in the 350-ms than in the 1,000-ms SOA was observed in both experiments. This can be explained by an attentional cuing effect elicited by the presentation of a temporally close auditory cue (McDonald, Teder-Sälejärvi, & Hillyard, 2000).

That naturalistic sounds elicited the cross-modal semantic priming effect faster (i.e., at the shorter SOA) than spoken words did suggests that the time required to access meaning for the former is shorter (Chen & Spence, 2013; Cummings et al., 2006; Saygin et al., 2005). Consistent evidence comes from the results of ERPs studies: For instance, Murray, Camen, Andino, Bovet, and Clarke (2006) have demonstrated that the brain activities associated with naturalistic sounds produced by living versus nonliving things can be discriminated around 70 ms to 119 ms after sound onset. The component associated with the meaning of spoken words (the N400; Kutas & Hillyard, 1980), on the other hand, typically starts 200 ms after word onset, and it could be delayed if the word is longer or else shares initial syllables with other words (van Petten, Coulson, Rubin, Plante, & Parks, 1999).

These results may partly be attributed to the nature of the acoustic signals that are associated with each type of stimulus: Naturalistic sounds associated with different object categories have distinct time-frequency spectrums from each other (e.g., Murray et al., 2006). By contrast, spoken words become comprehensible when the acoustic signals are abstracted into various phonetic representations, and the latter are then used to access their associated lexical representations (Obleser & Eisner, 2009). Consequently, a semantic network suggests that naturalistic sounds and visual pictures access semantics directly, whereas spoken words access their meanings via lexical representations (Chen & Spence, 2011, 2017; Glaser & Glaser, 1989). Hence, the cross-modal semantic interactions between naturalistic sounds and pictures would be expected to occur more rapidly than between spoken words and pictures, as demonstrated in the present study.

At the 1,000-ms SOA, only spoken words but not naturalistic sounds gave rise to cross-modal semantic priming effects, thus suggesting that the effect induced by naturalistic sounds is short-lived (see also Chen & Spence, 2017; Kim, Porter, & Goolkasian, 2014, when using the picture categorization task). Given that naturalistic sounds can access their meaning rapidly (within 350 ms in the current study), the short-lived priming effect suggests that the activated meaning would be forgotten rapidly as well, unless the information can be temporally maintained. The maintenance of representations of naturalistic sounds, nevertheless, is underpinned by the auditory imagery capability, or else by being transferred into lexical codes and stored in the phonological loop (Snyder & Gregg, 2011; Soemer & Saito, 2015), and both processes take extra time or cognitive resources. In contrast, spoken words essentially have the benefit of being maintained in the phonological loop in the working memory system (Baddeley, 2012), thus leading to the significant priming effect over a greater range of SOAs than naturalistic sounds (current study; Chen & Spence, 2017).

The final contrast lies in the fact that the time course of the cross-modal semantic priming effect by naturalistic sounds was stable, whereas that elicited by spoken words was modulated by the prior context (i.e., the order in which the SOAs were tested). Audiovisual integration/interactions involving speech sounds have been demonstrated to be flexible. For example, the cross-modal semantic priming effect by spoken words can be speeded up so as to be observed at around 350-ms SOA if the participants have been exposed to the longer SOA condition (the current study) or if the participants have to identify the target picture by reporting its name (Chen & Spence, 2011). In addition, the integration of verbal cues and visual lip movements occurs more often (indexed by a larger McGurk effect; McGurk & McDonald, 1976) if a series of congruent (compared to incongruent) audiovisual speech stimuli were presented beforehand (Nahorna, Berthommier, & Schwartz, 2012). Finally, the digits and letters that are presented subliminally to both vision and audition would be integrated only if the participants had consciously experienced these pairings prior to the test (Faivre, Mudrik, Schwartz, & Koch, 2014). The higher flexibility of audiovisual interactions involving spoken words than naturalistic sounds at the semantic level perhaps stems from the former accessing the semantic representations at an abstract, categorical, and modality-insensitive level, whereas the latter served as modality-specific and context-dependent attributes associated with the object cross-modally (Edmiston & Lupyan, 2015; Waxman & Gelman, 2009).

Together, the results of the two experiments reported here demonstrate that naturalistic sounds elicit more rapid cross-modal priming than do spoken words, which is likely determined by their speed of semantic access stemming from the different processing routes. On the other hand, the advantage of spoken words over naturalistic sounds to prime visual pictures across a more prolonged prime-target interval should result from the former being better maintained in working memory. Finally, consistent with previous studies, interactions between spoken words and visual signals are flexible—that is, they can be enhanced or inhibited by prior context or by task demands.

## Appendix

The auditory stimuli used in the present study. Note that the lengths of the naturalistic sound and spoken word referring to the same picture were matched at 350 ms for the 14 one-syllable words, 450 ms for the seven two-syllable words, and 500 ms for the three- (or more) syllable words (three words). The identification accuracy, confidence, and familiarity ratings (maximum score = 7) for the sounds reflect the mean performance of 18 participants (four males, mean age 28 years, reported in Chen & Spence, 2011). The ratings of imagery concordance (maximum score = 5) were acquired via an online study (30 participants for naturalistic sounds, 18 males, mean age 31 years; 30 participants for spoken words, 19 males, mean age 33 years). All scores were lower for naturalistic sounds than for spoken words. Identification accuracy: *t*(23) = 5.42, *p* < .001; confidence rating: *t*(25) = 8.33, *p* < .001; familiarity rating: *t*(26) = 8.38, *p* < .001; imagery concordance: *t*(24) = 4.24, *p* < .001; two-tailed, unequal variance assumed

**Table:**

			Naturalistic sounds				Spoken words			
Picture(Spoken word)	Naturalistic sound	Duration (ms)	Identification accuracy (%)	Mean confidence rating	Mean familiarity rating	Mean imagery concordance	Identification accuracy (%)	Mean confidence rating	Mean familiarity rating	Mean imagery concordance
Bird	Bird chirping	350	77.8	4.7	4.8	4.4	100	6.9	6.9	4.7
Cat	“Meow”	350	100	6.2	6.3	4.4	100	7.0	7.0	4.8
Cow	“Moo”	350	88.9	6.4	6.3	4.8	100	6.9	7.0	4.8
Dog	“Woof”	350	94.4	6.4	6.4	4.3	100	6.9	6.9	4.8
Duck	“Quack”	350	100	6.8	6.3	4.5	100	6.9	6.9	4.8
Eagle	Eagle call	450	55.6	4.9	4.6	4.5	100	6.8	6.9	4.8
Elephant	Roar	500	61.1	4.8	4.6	4.7	100	6.9	6.9	4.9
Frog	“Ribbit”	350	83.3	5.1	5.1	4.2	100	7.0	7.0	4.9
Goat	“Baa-baa”	350	72.2	5.6	5.4	4.4	100	6.9	6.7	4.9
Horse	Horse neighing	350	61.1	5.2	4.9	4.0	94	6.2	6.7	4.9
Pig	“Oink”	350	50.0	5.2	5.4	4.2	100	6.9	7.0	4.9
Rooster	Crowing sound	450	88.9	5.8	5.6	4.7	100	6.9	6.2	4.8
Car	Car starting	350	77.8	5.4	5.8	4.0	100	7.0	7.0	4.6
Door	Creak	350	88.9	4.8	5.2	4.3	100	6.9	6.8	4.7
Drum	Banging of a drum	350	94.4	5.8	5.8	4.3	100	7.0	6.8	4.8
Guitar	Guitar sound	450	88.9	6.0	6.4	4.8	100	7.0	6.9	4.9
Gun	Gunshot	350	61.1	4.3	4.1	3.6	100	6.9	6.9	4.8
Motorcycle	“Vroom-vroom”	500	33.3	4.8	5.1	3.4	100	6.9	6.7	4.7
Piano	Piano sound	450	94.4	6.1	6.2	4.8	100	7.0	6.9	4.7
Scissors	Clipping scissors	450	22.2	3.2	3.8	3.7	100	6.9	6.9	4.9
Switch	Click	350	27.8	3.8	4.2	2.0	100	6.6	6.5	4.6
Telephone	Telephone ringing	500	100	6.6	6.7	4.7	100	7.0	7.0	4.6
Trumpet	Trumpet sound	450	83.3	5.3	5.3	4.6	100	7.0	6.7	4.9
Whistle	Whistling	450	66.7	5.4	5.5	4.8	94.4	6.8	6.6	4.9
	Mean (*SEM*)	60.9 (7.9)	5.2 (0.2)	5.2 (0.2)	4.3 (0.1)	98.7 (0.9)	6.9 (0.1)	6.8 (0.1)	4.8 (0.02)	

The stimuli used in the practice sessions were fly (humming fly), tiger (tiger roaring), bell (bell ringing), and cannon (cannon fire)

## Footnotes

1 The interstimulus interval was 400, 1000, or 1500 ms in Lupyan and Thompson-Schill (2012). However, since they did not report the duration of the auditory cue, it is not possible to determine the corresponding SOAs. Nevertheless, the SOAs were certainly longer than the 346 ms in Chen and Spence (2011).
